# Computational Insights on Sulfonamide Imprinted Polymers

**DOI:** 10.3390/molecules13123077

**Published:** 2008-12-10

**Authors:** Chartchalerm Isarankura-Na-Ayudhya, Chanin Nantasenamat, Prasit Buraparuangsang, Theeraphon Piacham, Lei Ye, Leif Bülow, Virapong Prachayasittikul

**Affiliations:** 1 Department of Clinical Microbiology, Faculty of Medical Technology, Mahidol University, Bangkok 10700, Thailand; 2 Pure and Applied Biochemistry, Chemical Center, Lund University, Box 124, 22100 Lund, Sweden

**Keywords:** Molecular imprinting, Molecularly Imprinted polymer, Sulfonamide, Molecular modeling

## Abstract

Molecular imprinting is one of the most efficient methods for preparing synthetic receptors that possess user defined recognition properties. Despite general success of non-covalent imprinting for a large variety of templates, some groups of compounds remain difficult to tackle due to their structural complexity. In this study we investigate preparation of molecularly imprinted polymers that can bind sulfonamide compounds, which represent important drug candidates. Compared to the biological system that utilizes metal coordinated interaction, the imprinted polymer provided pronounced selectivity when hydrogen bond interaction was employed in an organic solvent. Computer simulation of the interaction between the sulfonamide template and functional monomers pointed out that although methacrylic acid had strong interaction energy with the template, it also possessed high non-specific interaction with the solvent molecules of tetrahydrofuran as well as being prone to self-complexation. On the other hand, 1-vinyl-imidazole was suitable for imprinting sulfonamides as it did not cross-react with the solvent molecules or engage in self-complexation structures.

## Introduction

Molecular imprinting is one of the most efficient methods for preparing synthetic receptors that possess user-defined recognition properties [1,2,3]. Despite that many bioactive compounds have been used as templates to prepare highly selective polymers for applications in separation [4], catalysis [2], assay and sensing purposes [5], there are certain chemical entities that are difficult to tackle because of their intrinsic structural complexity. Frequently, these compounds are encountered in natural products and in the drug development process. It is therefore important to investigate molecular imprinting for these types of compounds, because the imprinted polymers are potentially useful as artificial receptors for screening of combinatorial product libraries [6,7,8] and for target-directed organic synthesis [9,10,11].

The molecular imprinting of sulfonamide compounds via bulk polymerization has previously been reported by Zheng *et al*. in a series of works on the antibiotics sulfamethazine and sulfamethoxazole [12,13,14]. In their studies methacrylic acid, 4-vinylpyridine, acrylamide and their mixtures were demonstrated to be good functional monomer(s) for these sulfonamide compounds. In a relevant study by Chen *et al*. [15], a uniformly-sized molecularly imprinted polymer against sulfamethazine was produced using the one-step swelling and polymerization method and was shown to give good binding performance. Drug delivery devices based on imprinted polymers against the colon prodrug, sulfasalazine, were successfully demonstrated by Puoci *et al*. [16].

**Figure 1:**
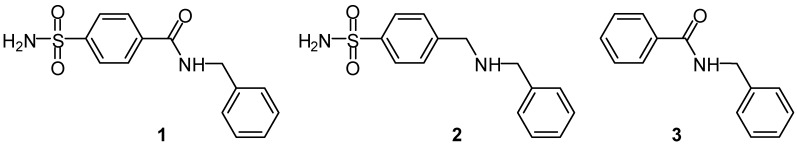
Chemical structure of the template molecule **1** and rebinding analogues **2** and **3**.

Computational approaches based on quantum chemical techniques have been extensively utilized in recent years to explore and select optimal functional monomers for any given template molecule of interest. Among the first reported work utilizing molecular mechanics was that performed by Takeuchi *et al*. [17] and Yoshida *et al*. [18]. The research group of Piletsky and collaborators [19,20,21,22,23,24,25,26,27,28] pioneered and demonstrated in their series of work on the feasibility of using the molecular mechanics-based Leapfrog algorithm to screen from a virtual library of functional monomers followed by selecting the optimal functional monomer based on the best interaction energy. Moreover, we have previously proposed the use of charge-based descriptors with artificial neural networks as a novel approach in establishing quantitative structure-imprinting factor relationship models [29,30]. The sulfonamide compounds investigated in this study (Figure 1) encompass important carbonic anhydrase inhibitors that are potentially useful as drug candidates.

In general, sulfonamide compounds have poor solubility in standard imprinting solvents, and are difficult to tackle due to their lack of functional groups that can be exploited. In this study we intended to prepare molecularly imprinted polymers using a “rational design” approach, where we utilize the hydrogen bond interactions between template and functional monomers. Unlike the biological system, multiple hydrogen bond interactions are found to be responsible for the obtained imprinting effect in a polar organic solvent. The mechanism of interaction between template and functional monomers is essential toward the understanding of the binding performance of the imprinted polymers; therefore, molecular modeling was employed to study the binding conformation and interaction strength of template and functional monomer molecules in relation to the observed imprinting effect.

## Results and Discussion

### Molecular imprinting using MAA as functional monomer

Methacrylic acid (MAA) has been used as a universal functional monomer to prepare imprinted polymers against both weakly acidic [31] and basic molecules [32] employing hydrogen bond interactions, because it can act as both a hydrogen bond donor and acceptor. When sulfonamide **1** was used as template, it could be dissolved in THF at a relatively low concentration in the presence of MAA.

The imprinted polymer (**MIP 1a**) and non-imprinted polymer (**NIP 1a**) were prepared and subjected to solvent extraction to remove the template, and binding property of the polymers tested in THF. When 20 mg of polymer was incubated in 1 mL of **1** in THF at a concentration of 250 µg mL^-1^, the amount of template bound was found to be 9.6% for **MIP 1a** and 3.8% for **NIP 1a**, which showed a definite difference between the imprinted and the non-imprinted polymer. However, the total binding in THF was quite limited due to the rather weak interactions. This result is in line with a study carried out by Dirion *et al*. [33], where the weakly acidic estradiol template generated low imprinting effect when MAA was used as the functional monomer in THF. Under the specific condition, THF may interfere with the MAA-**1** interaction by acting as a competing hydrogen bond acceptor, therefore a stronger hydrogen-accepting monomer (general base) is needed to increase the binding efficiency for the present sulfonamide template.

### Molecular imprinting using VIM as functional monomer

Solubility of sulfonamide **1** in THF was greatly enhanced when 2 equivalents of 1-vinylimidazole (VIM) were added. To verify that VIM indeed formed stable complex with **1**, we added increasing amount of VIM into a solution of **1** dissolved in THF, and followed the change in the proton NMR signals arising from the template. When the molar equivalents of VIM were increased from 2 to 4, the sulfonamide and the amide signal of **1** displayed a pronounced downfield shift of 0.32 ppm and 0.26 ppm, respectively, which indicated formation of stable complex between the template and the functional monomer via the two distinct interacting points. Due to the difficulty of dissolving template at low VIM doses, quantification of the association constant and binding stoichiometry was not performed. Instead, a 1:4:4 molar ratio of template:VIM:TRIM was directly used to prepare an imprinted polymer (**MIP 1b**). Binding properties of the imprinted polymer were tested in THF using equilibrium binding analysis. When 20 mg of polymer was used, the percentage of **1** bound to **MIP 1b** was approximately 2 times that of **NIP 1b**, indicating that the template-functional monomer complex formed in the pre-polymerization solution facilitated generation of the specific binding sites in the imprinted polymer. In the present system, VIM alone, as a hydrogen bond acceptor, was able to form hydrogen-bonded complex only with the sulfonamide template. No self-complexation structure from VIM (such as the MAA dimer formed in aprotic solvent) existed, therefore the non-imprinted polymer **NIP 1b** contained statistically distributed functional imidazole group. Hence, template binding to **NIP 1b** should be a result of the sole contribution of non-specific adsorption.

**Figure 2:**
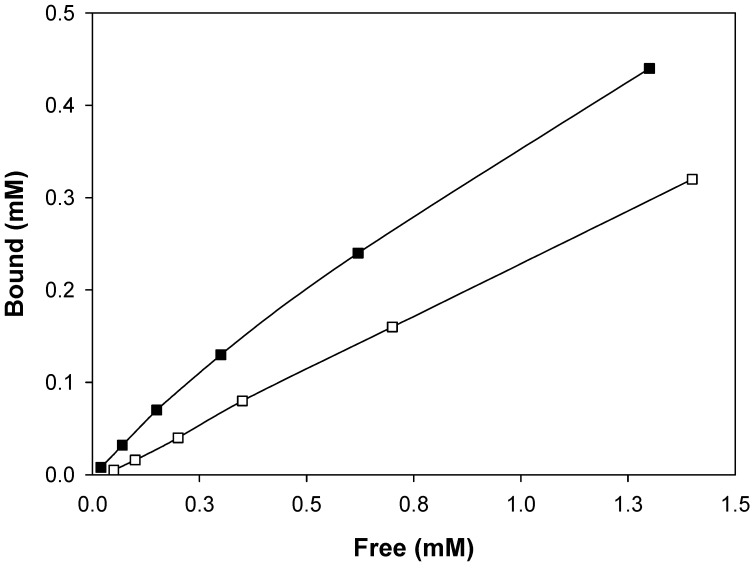
Binding isotherm of **MIP 1b** (■) and **NIP 1b** (□) obtained by titration of the polymers with the template sulfonamide, **1**. The binding data for **NIP 1b** was fitted using linear regression to give the nonspecific constant (*NS*), which was subsequently fixed to calculate the apparent dissociation constant (*K*_D_) and the number of specific binding sites (*B*_max_) for **MIP 1b** using the following equation: *B* = *F* × *B*_max_ / (*F* + *K*D) + *NS* × *F*.

Using an increasing amount of template to titrate a fixed amount of polymer in THF, we established binding isotherms for both **MIP 1b** and **NIP 1b** (Figure 2). When **NIP 1b** was titrated, a linear isotherm was obtained, which represented typical non-specific adsorption of analyte associating to isolated functional groups. The non-specific binding constant (*NS* = 0.22) could be obtained by analyzing the binding data on the control polymer (**NIP 1b**) using a linear regression curve fit. The *NS* was then used to fit the binding data obtained on the imprinted polymer (**MIP 1b**) using a non-linear regression program, where the specific binding sites were assumed to be independent from one another. This generated an apparent dissociation constant of 1.1 mM, and binding site population of 15 µmol g^-1^ for the imprinted polymer (**MIP 1b**).

In the present system we ended up with a relatively high non-specific binding, which was possibly caused by the lack of extra binding moiety in the target template besides the sulfonamide and the amide groups. Our attempt to utilize divinylbenzene as cross-linker to provide π–π interaction with the template in THF failed, as addition of this cross-linker caused template precipitation. The importance of the sulfonamide group for selective binding is reflected by the binding results with compound **3**, which is identical to the original template except for the lack of the sulfonamide group. Under the same condition, the amount of **3** bound to the imprinted (**MIP 1b**) and non-imprinted polymer (**NIP 1b**) were almost identical (14%) and were much lower than that of the sulfonamide compound **1**. The binding selectivity of **MIP 1b** can be further verified by comparing its analyte response using a test compound having different functionalities. Compared to **1**, compound **2** was found to bind to **MIP 1b** and **NIP 1b** more efficiently, presumably due to the stronger hydrogen bond donor effect from the amine moiety (data not shown). However, no differential binding could be noticed between **MIP 1b** and **NIP 1b** (see the selectivity index calculated in Table 1), indicating that the polymer imprinted against **1** did not cross-react to **2**. The cross recognition of homologous molecules by molecularly imprinted polymers has been reported in earlier literature [11,34,35]. These imprinted polymers are considered highly valuable as artificial receptors for library screening and separation of a group of interesting molecules.

**Table 1:** Preparation and characterization of molecularly imprinted polymers using template **1**.

*Polymer*	*1 (mmol)*	*Functional monomer (mmol)*		*TRIM* *(mmol)*	*Selectivity index (SI)^a^*		
		MAA	VIM		1	2	3
**MIP 1a**	0.6	2.4	―	24	2.7	n.d. **^b^	n.d. **^b^
**NIP 1a**	―	2.4	―	24	―	―	―
**MIP 1b**	4.8	―	19.2	19.2	1.8	1.1	1.0
**NIP 1b**	―	―	19.2	19.2	―	―	―

^a^ Selectivity index is the ratio of partition coefficient of an analyte on the imprinted polymer in relation to that on the non-imprinted polymer, and calculated according to equation: *SI* = (*B*/*F*)_MIP_ / (*B*/*F*)_NIP_. *B* and *F* are concentration of the test compound that binds to the polymer and those that are free in solution, and are determined in equilibrium binding experiment using HPLC quantification. For *SI* evaluation, a fixed amount of polymer (20 mg) and test compound (250 µg) were incubated in 1 mL of THF; ^b^ n.d. : Not determined.

### Computer simulation

**Figure 3:**
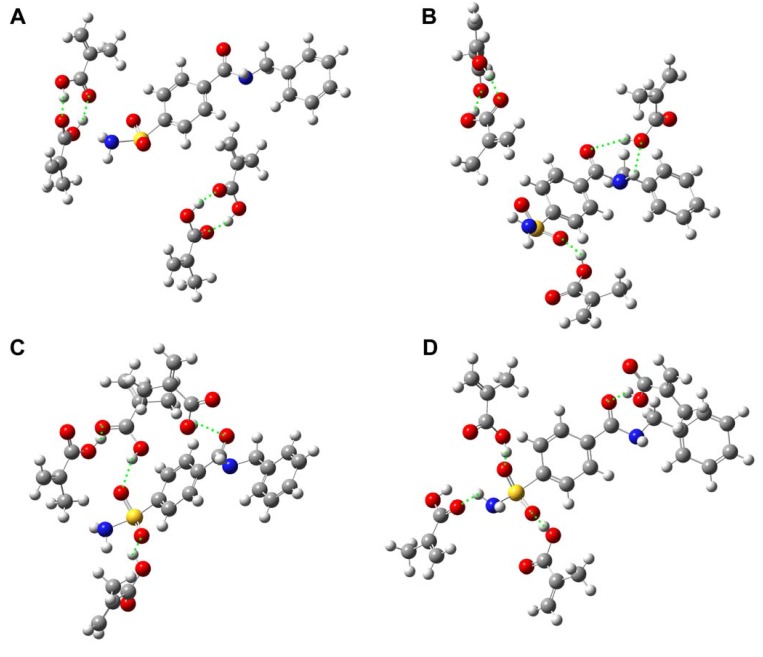
Sampling of some of the possible modes of interaction of template **1** with MAA. Two MAA dimers with no interaction with template (A), one MAA dimer formation and two MAAs interacting with template (B), incomplete MAA dimer formation interacting with template with two MAA interacting with template (C), and four MAA interacting with template. Other possible conformations that may exist are not shown. Hydrogen bond interactions are represented by green dotted lines.

**Figure 4:**
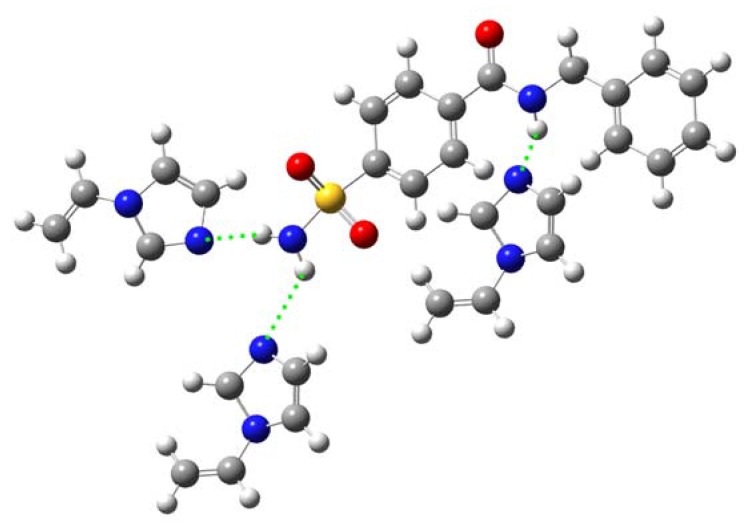
Proposed mode of interaction of template **1** with VIM via two H-bondings, particularly the interaction of nucleophilic imidazole N-atom of VIM with amino group of sulfonamide and the H-bonding of imidazole N-atom of VIM with central amide NH.

Sampling of the possible modes of interaction for sulfonamide template **1** with functional monomers, MAA and VIM, are shown in Figure 3 and Figure 4, respectively. It can be seen in Figure 3 that MAA can interact with template **1** in many possible ways. The four possible modes of interaction as shown in Figure 3 are only a few examples of **1**-MAA complexation. On the contrary, it is shown in Figure 4 that VIM can interact with template **1** at a molar ratio of 3:1 with three hydrogen bond donors of the central amide and amino group of sulfonamide present on template **1**. These observations suggest that the imprinted polymer of **1** is likely to have heterogeneous binding sites when interacting with MAA. This can be attributed to the fact that MAA can act as both hydrogen bond donor and acceptor and can therefore interact with the template at one or two points of interaction as well as form MAA dimers. On the other hand, interaction with VIM would give a more homogeneous binding site as illustrated by the fewer possible modes of interaction.

To save computational cost, calculations of the interaction energy (Table 2) were derived from a 1:1 template-monomer complex. It is observed that the interaction energy of 1:1 and 1:2 template-monomer complexes, although of different magnitude, exhibited no difference in the general trend of interaction strength of **1**-MAA or **1**-VIM complexes. Keeping in mind the simplification of the interaction energies calculated from 1:1 template-monomer complex to represent those of 1:4 complex (as performed experimentally), both are expected to give similar trends as far as interaction energies are concerned. This notion is supported by our previously reported theoretical calculations [36] as well as those of Diñeiro *et al*. [37,38].

**Table 2:** Interaction energies of **1** with MAA or VIM.

	*E (a.u.)*	*ΔE (a.u.)^a^*	*ΔE (kJ/mol)^b^*
**1**	–1,275.2206		
MAA	–306.4671		
VIM	–303.6062		
THF	–232.4475		
**1**-MAA 1^c^	–1,581.7052	–0.0175	–45.8398
**1**-MAA 2^d^	–1,581.7227	–0.0350	–91.9364
**1**-MAA 3^e^	–1,581.7112	–0.0235	–61.7582
**1**-MAA 4^f^	–1,581.7038	–0.0160	–42.1389
**1**-MAA 5^g^	–1,581.7035	–0.0158	–41.4375
**1**-VIM 1^h^	–1,578.8458	–0.0190	–49.9639
**1**-VIM 2^i^	–1,578.8427	–0.0158	–41.5927
MAA-MAA	–612.9857	–0.0514	–135.0195
THF-MAA	–538.9426	–0.0279	–73.2438
THF-VIM	–536.0615	–0.0077	–20.1504

^a^Δ*E* is the interaction energy derived from equation 1; ^b^Δ*E* is converted from a.u. to kJ mol^-1^ by multiplying the energy value in a.u. by the conversion factor 2.626 x 10^3^; ^c^ Figure 6A; ^d^ Figure 5A; ^e^ Figure 6B; ^f^ Figure 5B; ^g^ Figure 6C; ^h^ Figure 7A; ^i^ Figure 7B

**Figure 5:**
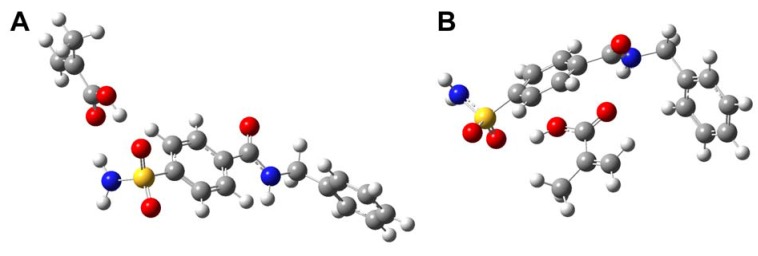
Geometrically optimized structures of MAA and template **1** in a two point interaction through H-bonding with carboxylic acid of MAA with the S=O and NH sulfonamide moieties (A) and H-bonding with carboxylic acid of MAA with the sulfonyl O-atom and NH of central amide moieties (B).

Based on our computer simulation with the *ab initio* quantum chemical techniques, it is found that MAA was able to interact with both hydrogen bond donors and acceptors of sulfonamide template **1**. On the contrary, VIM can only interact with the hydrogen bond donor of template **1**. This is to be expected since MAA can act as both hydrogen bond donor and acceptor while VIM can only behave as hydrogen bond acceptor. As a result, the inherent property of MAA allows it to engage in two points of interaction, particularly, with both hydrogen bond donor and acceptor of template **1** simultaneously as shown by our simulation (Figure 5). It was shown that two point interaction of MAA with template **1** at the sulfonamide group (Figure 5A) gave higher interaction energy value than two point interaction at the sulfonamide and central amide group (Figure 5B) as illustrated by the interaction energy value of –91.9364 and –42.1389 kJ mol-1, respectively.

**Figure 6:**
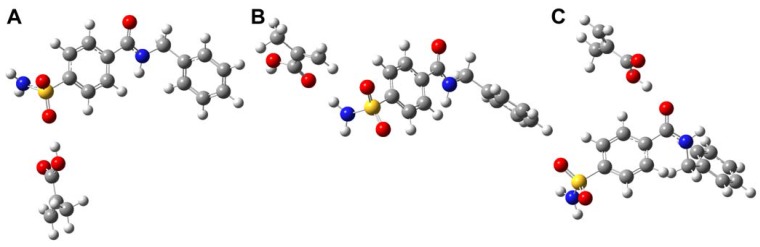
Geometrically optimized structures of MAA and template **1** calculated at HF/3-21G(d) level in a one point interaction via the H-bond of sulfonyl O-atom with OH of carboxylic acid (A), H-bonding of amino sulfonyl with carbonyl O-atom of MAA (B), H-bond of carbonyl O-atom of amide with OH of carboxylic acid moieties.

**Figure 7:**
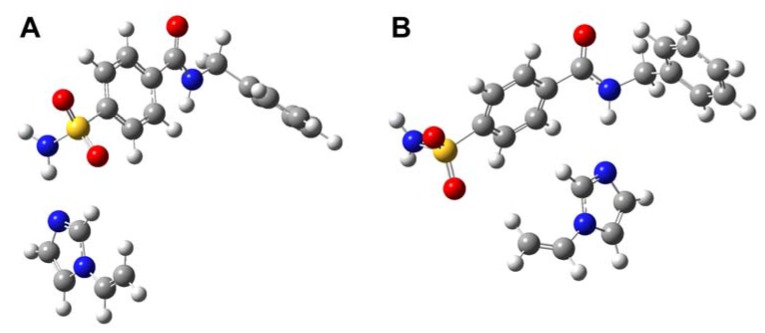
Geometrically optimized structures of VIM and template **1** calculated at HF/3-21G(d) level in a one point interaction via the H-bond of imidazole N-atom of VIM with NH of sulfonamide (A) as well as the H-bond of imidazole N-atom of VIM with NH of central amide moieties (B).

Our calculations revealed that the interaction energy of template-monomer complexations is greater at the sulfonamide group of template **1** for MAA, as observed by the interaction energy values of –45.8398 (Figure 6A) and –61.7582 kJ mol-1 (Figure 6B). Likewise, strong interaction with the sulfonamide moiety gave interaction energies of –48.9565 and –49.9639 kJ mol^-1^ for VIM based complexes as illustrated in Figure 7A. On the other hand, low interaction energy values of –41.4375 and –41.5927 kJ mol^-1^ were observed for MAA (Figure 6C) and VIM (Figure 7B) based complexes, respectively.

These results suggest that the functional monomers interact more strongly at the sulfonamide moiety than with the central amide of template **1**. This is further supported by the experimental results with compound **3** which displayed no particular preference for imprinted polymers over non-imprinted polymers as its binding capacity were almost identical for both imprinted (**MIP 1b**) and non-imprinted (**NIP 1b**) polymers. On the other hand, there is pronounced imprinting effect for template **1**, which could be attributed to the presence of the sulfonamide group. Compound **3** is identical to **1** except for the lack of the sulfonamide moiety, suggesting the importance of the sulfonamide group in molecular recognition with basic functional monomers [13,14], particularly VIM, in our study.

It was previously mentioned that the MAA system suffered from interference by solvent molecules of THF and the propensity toward MAA dimer formation, both of which may severely impede its binding performance. To investigate such effects, molecular models of each scenario were constructed and geometrically optimized at HF/3-21g(d) level followed by single point calculation at B3LYP/6-31g(d) level. As presented in Table 2, results indicated that the interaction energy of MAA dimer was the strongest of all at –135.0195 kJ mol^-1^. The energy of interaction for THF with MAA was ranked third at –73.2438 kJ mol^-1^ while the corresponding value for THF with VIM were found to be the least of all at –20.1504 kJ mol^-1^. The former supports the notion that solvent molecules of THF hinder the binding performance of MAA-based polymers, while the latter pointed out that THF had minimal effect on VIM-based polymers. In an excellent review by Karim *et al*. on the selection criteria of functional monomers, it was suggested that the choice of monomer should not be confined only to the interaction energy of template-monomer adducts and that attention should also be placed on other aspects such as solubility, stability, and reactivity of the monomer and its pre-polymerization mixture [39].

## Conclusions

This study reports the synthesis of sulfonamide imprinted polymers and the utilization of computer simulation in elucidating the mechanistic details governing the polymer’s recognition ability. Rebinding results indicated that MAA-based polymers possessed slightly higher selectivity than VIM-based polymers. However, there are certain drawbacks which limit the potential application of MAA system: i) propensity of MAA to form self-complexation structure, ii) interference from THF as competing hydrogen bond acceptor, iii) template insolubility, and iv) the multitude of possible molecular interaction between sulfonamide template with MAAs contributes to binding site heterogeneity. Hence, VIM was the suitable functional monomer as they do not possess any of these deficiencies as well as accommodating higher amounts of sulfonamide templates owing to their better template solubility. The validity of these points was addressed in a series of computer simulations and their calculated interaction energy further supported those notions. In a future study, we will exploit the imidazole-amine interaction between VIM and sulfonamide templates that are similar to **2**, and expect to obtain improved binding affinity and selectivity. A comparative study of other H-bond acceptors as potential functional monomers is also worthy to consider in forthcoming studies.

## Experimental

### General

*N*-(4´-sulfamoylbenzoyl)benzylamine (**1**) [40] and 4-(benzylaminomethyl)-benzenesulfonamide (**2**) [41] were prepared following literature methods. 4-Carboxybenzenesulfonamide, benzylamine, *N*-benzylbenzamide (**3**), Raney® 2800 nickel (hydrogenation catalyst slurry in water, Ni ≥89%, Al ≥6-9%, pH >9.5), sodium borohydride, methacrylic acid (MAA), 1-vinylimidazole (VIM), ethylene glycol dimethacrylate (EDMA), trimethylolpropane trimethacrylate (TRIM) and azobis-isobutyronitrile (AIBN) were purchased from Aldrich. 4-Cyanobenzene-1- sulfonamide was obtained from Maybridge plc (Cornwall, England). NMR measurements were carried out on a 400 MHz Bruker NMR spectrometer.

### NMR titration

*N*-(4´-sulfamoylbenzoyl)benzylamine (**1**, 133 mg, 0.458 mmol), was dissolved in a series of solutions containing VIM (83 mg, 0.916 mmol; 125 mg, 1.37 mmol; 166 mg, 1.83 mmol) in 750 µL of deuterated tetrahydrofuran (THF). NMR spectra for the samples were collected, from which the proton chemical shift of the sulfanomide and of the amide were calibrated against the solvent signal at δ 1.73 ppm.

### Molecularly imprinted polymers using 1 as template

Molecularly imprinted polymers (**MIP 1a** and **MIP 1b**) were prepared in anhydrous THF using **1** as the template (Table 1). The template was dissolved in anhydrous THF (7.87 mL) containing the functional monomer (MAA or VIM). To the solution was then added the cross-linker, TRIM and AIBN (158 mg). The obtained solution was transferred into a 20 mL screw-caped borosilicate tube and purged with argon for 5 min. The tube was then submerged in a 60°C water bath for 12 h. The solid polymer was broken up and ground with a mechanical mortar. Particles with apparent diameter of 10-25 µm were collected by repetitive sieving and sedimentation in acetone. To remove the template, methanol containing 10% acetic acid (v/v) was used for extraction. Quantitative removal of template was ensured by monitoring the amount of template remained in the extraction solvent by HPLC analysis. The non-imprinted control polymers (**NIP 1a** and **NIP 1b**) were prepared in the same way as used for the corresponding imprinted polymers, except that the template was omitted during polymerization.

### Binding analysis

Equilibrium binding analysis was carried out by incubating fixed amount of polymers in a 1 mL volume of analyte solution at ambient temperature for 12 h. A rocking table was used to provide gentle mixing. After the incubation, samples were centrifuged at 12 000 rpm for 10 min, from which 0.75 mL supernatant was collected for determination of the free analyte by HPLC analysis.

### HPLC analysis

Reverse phase HPLC analysis was carried out using a LaChrom L-7100 solvent delivery system, a L-7455 diode array detector and a D-7000 HPLC System Manager software package (Merck KgaA, Darmstadt, Germany). A Chromolith Performance column (RP-18 e) from Merck (Darmstadt, Germany) was used, applying a mobile phase of water: acetonitrile = 70: 30 (v/v, both containing 0.1% TFA) at a flow rate of 1.0 mL min^-1^. The analyte was monitored at 230 nm. For each analysis 10 µL of sample was injected.

### Computer simulation

Sulfonamide templates and functional monomers were drawn using GaussView, version 3.09 [42]. The functional monomers were placed near the functional groups of the sulfonamide template **1**. Next, template **1**, functional monomers (MAA and VIM), and their complexations were subjected to full geometry optimization under Gaussian 03W [43] at the Hartree-Fock level of theory using the 3-21G(d) basis set. This quantum chemical calculation provided insights on possible modes of interaction for the template-monomer complex. The aforementioned procedures were repeated for all putative points of interaction. The template-monomer interaction energy was calculated from the following equation:

(3) $$\Delta E=\left|E_{template-monomer}-E_{template}-E_{monomer}\right|$$

where Δ*E* is the interaction energy, *E_template-monomer_* is the energy of template-monomer complex, *E_template_* is the energy of template molecule, and *E_monomer_* is the energy of functional monomer molecules. The energy values were derived from single point calculation at the Density Functional Theory (DFT) using Becke’s three-parameter Lee-Yang-Par (B3LYP) functional and 6-31G(d) basis set.
